# Socio-Environmental and Hematological Profile of Landfill Residents (São Jorge Landfill–Sao Paulo, Brazil)

**DOI:** 10.3390/ijerph14010064

**Published:** 2017-01-11

**Authors:** Vivianni Palmeira Wanderley, Fernando Luiz Affonso Fonseca, André Vala Quiaios, José Nuno Domingues, Susana Paixão, João Figueiredo, Ana Ferreira, Cleonice de Almeida Pinto, Odair Ramos da Silva, Rogério Alvarenga, Amaury Machi Junior, Eriane Justo Luiz Savóia, Rodrigo Daminello Raimundo

**Affiliations:** 1 Environmental Health Management Department, ABC MedSchool, Santo André 09060-650, SP, Brazil; profferfonseca@gmail.com (F.L.A.F.); cleo.almeidaabc@hotmail.com (C.d.A.P.); oramos2002@ibest.com.br (O.R.d.S.); alvarengaro@gmail.com (R.A.); machijr@yahoo.com.br (A.M.J.); erianejusto@yahoo.com.br (E.J.L.S.); rodrigodaminelloraimundo@usp.br (R.D.R.); 2 Biological Sciences Department, Institute of Environmental, Chemistry and Pharmaceutical Sciences, Federal University of São Paulo, Diadema 04060-650, SP, Brazil; 3 Environmental Health Sciences Department, IPC, EsTesC, Coimbra Health School, Coimbra 3046-854, Portugal; andrequiaios@gmail.com (A.V.Q.); zee_domingues@hotmail.com (J.N.D.); supaixao@estescoimbra.pt (S.P.); laboratoriofmabc@yahoo.com.br (J.F.); anaferreira@estescoimbra.pt (A.F.)

**Keywords:** landfill, waste, socio-environmental impact, hematologic diseases

## Abstract

We are experiencing an unprecedented urbanization process that, alongside physical, social and economic developments, has been having a significant impact on a population’s health. Due to the increase in pollution, violence and poverty, our modern cities no longer ensure a good quality of life so they become unhealthy environments. This study aims to assess the effect of social, environmental and economic factors on the hematologic profile of residents of Santo André’s landfill. In particular, we will assess the effect of social, economic, and environmental factors on current and potential disease markers obtained from hematological tests. The research method is the observational type, from a retrospective cohort, and by convenience sampling in Santo André in the Greater ABC (municipalities of Santo André, São Bernardo do Campo and São Caetano do Sul, southeast part of the Greater São Paulo Metropolitan Area, Brazil). The study determined a socio-environmental profile and the hematologic diseases screening related to a close location to the landfill. The disease manifests itself within a broad spectrum of symptoms that causes changes in blood count parameters. The objective of this work is to show that there is an association between social, environmental and economic factors and a variety of serious disease outcomes that may be detected from blood screening. A causal study of the effect of living near the landfill on these disease outcomes would be a very expensive and time-consuming study. This work we believe is sufficient for public health officials to consider policy and attempt remediation of the effects of living near a landfill.

## 1. Introduction

Nowadays, due to higher levels of pollution, violence and poverty, cities can no longer ensure a good quality of life for everyone and have become unhealthy environments for a great many local inhabitants [1]. An unprecedented urbanization, along with physical, social and economic developments, is having a significant impact on the health of the population [1,2].

Besides the continuous demographic growth, these factors have an implicit effect on the amount of resources that have to be consumed in order to sustain all citizens of the Earth. This increases waste production, which invariably affects society dynamics in general.

Landfill disposal remains the main destination in waste management, and it is expected to remain so for the next decades [3,4,5]. It is estimated that at the worldwide scale, over five million people die every year due to waste-related diseases [2]. The adverse effects of municipal solid waste on the environment, not to mention on public and individual health, are widely recognized by several authors [6], who point out a deficiency in the implemented systems and especially the lack of a strong policy emphasizing health safeguards [7].

A few authors state that societies all over the world are focused on consumption, and, therefore, there are significant losses of organic and inorganic materials: waste type [8,9], which would imply an uncertain future for Public Health. The improvement suggestion would be educating the population for a sustainable consumption and a strategic waste management system [9], such as: recycling and treating degraded soils; and to which extent these landfill sites would be usable.

The large amount of waste was not a concern for an extensive period due to the distance between disposal sites and urban areas. Meanwhile, with the population growth, it has been difficult to reduce the distance between them [10]. Another emerging problem linked to population growth is the unsystematic construction of homes in hazardous locations and quite vulnerable environmental areas, which usually lack urban infrastructure (sanitation, electricity), among other things [11,12].

It has been scientifically shown that residences on or near a landfill have impacts on the residents’ health and on the environment: air quality, water quality, and soil pollution. The effects of landfills are major public health issues. Consequently, waste, waste treatment and disposal are major sanitary and environmental concerns to cities [3,13,14].

The environmental degradation scenario is unquestionable, and there is a lack of policies to prevent and ameliorate the crisis. The complexity of analyzing all impacts demands studies on the various effects of waste produced daily by the population [15,16]. The first step is to identify any adverse health outcomes using blood tests, which can be an important tool for the evaluation of various situations, such as diagnosis and progression of hematologic diseases, detection of infections and therapeutic monitoring [17].

Therefore, a hemogram can guide the initial suspicions supported by clinical files. The importance of the hematologic analysis is assumed to be an important diagnosis tool, providing useful information for a better handling of these cases.

It is necessary to adopt a set of measures that include politics’ globalization, government social efficiency and social participation growth. It is the government’s duty to ensure that change is not only possible but always sustained in clear objectives as well in results well defined and with special properly defined interventions [18,19,20].

Santo André has an area of 174.38 square kilometers and is located in the Greater ABC (municipalities of Santo André, São Bernardo do Campo and São Caetano do Sul, southeast part of the Greater São Paulo Metropolitan Area, Brazil), 18 km from the capital city of São Paulo. The city is strategic for the logistics sector, as it lies in the main economic center of the country. Santo André has 678,486 inhabitants and is the tenth largest city in the state of São Paulo. The landfill of São Jorge (217,000 m^2^) is located in Santo André, and is surrounded by the slum São Jorge—about 1400 families. Every month, the landfill receives about 13 thousand tons of household waste and 250 tons of sterilized medical waste [11].

Around the landfill of São Jorge, there are signs that the environment has suffered both physical and social impacts [21,22]. The landfill began to operate in the 1980s and is located in the São Jorge District. Santo André’s landfill is a facility that provides treatment and disposal of solid waste produced in the municipality. Therefore, it evolved from a non-official dumping location to a controlled one with good environmental practices. Nowadays, it is an area destined to receive solid waste produced in Santo André and is the most suitable and sustainable destination for the waste.

This study aims to measure the socio-environmental and hematologic profile of residents of Santo André’s landfill—the “*Espírito Santo* suburbs”—by using the social and demographic data of the studied area and association between socio-environmental factors and the alterations in hematological parameters as precursors to diseases. This fieldwork will allow better urban planning and Public Health policies for residential areas on and near landfills, as well as a greater degree of social and environmental responsibility for all sectors of society.

## 2. Method

The research method is observational with a retrospective cohort study where the samples are obtained by convenience in Santo André in the Greater ABC region. The study consists of the elaboration of a demographic and environmental profile and assesses the prevalence of potential diseases identified by hematological screening that may be the result of housing conditions and/or exposure to environmental contaminants from the landfill. The study must be understood as a primary tracking diagnosis, and it will be the kick-off for a transversal study of *Espírito Santo* inhabitants that will be performed in the near future.

The exposed group lives in the vicinity of the landfill. There is a very little knowledge about the reality of this population due to the fact that national entities do not have permission to enter the area to study and help this community. Furthermore, this study got a special permit to develop a campaign for being the spin-off of a more transversal study. The community inhabits a place that was a dumping ground and it is actually contiguous to the landfill; therefore, the study’s aim was to establish connections among the location and the community’s health.

The study was conducted in two phases. The first phase consisted of a survey and research profile of the residents as the following variables: age, gender, type of house, water treatment, water supply and type of sewage, based on interviews and completing a questionnaire. The second phase consisted of the collection of blood samples into two distinct groups: a group of people living in the community (experimental group) and a group of random people attending a health facility located in the central city (control group).

According to the data obtained, a descriptive and comparative analysis of the differences in hematologic patterns between both groups was made.

The blood samples were collected through peripheral venopunction using the vacuum method. After recovery, the blood was added to the tube with EDTA. The samples were homogenized for 10 min and evaluated through flux cytometry with ABX Pentra 120 equipment (Horiba Medical, Montpellier, France). The serial evaluations were performed in blades using the Leishman method in order to obtain the procedures approached. The analysis was carried out according to good biomedical practices.

Quantitative and qualitative alterations in blood count (viral, bacterial and parasitic) were measured by reading lamina by Fernando Luiz Affonso Fonseca (hematologist)—a blood sample.

A total of 100 blood samples were harvested, but only 62 of them were considered biologically viable. The viability of the blood sample complied with good practices in clinical analyses. Thus, samples hemolyzed with jaundice or still lipemic were not included in the hematological analysis.

Therefore, in a group of inhabitants of the central region, 30 individuals were included (unexposed group). From the group of inhabitants of the vulnerable region (exposed group), 84 were included. However, from these, only 32 consented to having their blood harvested. 

The information gathering tool used in this study was a questionnaire taken from the database developed by SEMASA, the Bureau of Environmental Issues of Santo André, São Paulo, Brazil, the institute responsible for the approval of construction, basic sanitation and water supply, as well as for assessing the regularity of housing developments. The applicants were the work’s researchers, supervised by the head researcher of the University.

From a total of 100 questionnaires filled out, 84 were considered adequate (valid questionnaires). Thus, 16 were discarded, due to inadequacies in the information provided or a lack of consent from the individuals.

A descriptive analysis of all variables presented was performed in terms of their relative and absolute terms. In order to evaluate the association between the qualitative variables, a chi-square test was used, and/or the exact Fisher test. The level of significance was 5%. The data were processed and treated in the statistics software SPSS 21.0 (Released 2012, IBM SPSS Statistics for Windows, Version 21.0, IBM Corp., Armonk, NY, USA).

At last, an exact Fisher test was applied, in order to evaluate whether there is a connection between the hematological results and the type of exposure, as well as the quantity of hematological changes. The exact Fisher test is used to calculate the association of the analyzed features when the total amount of data is small and when the expected values in one or more of the cells of the 2 × 2 table is less than or equal to five. Thus, in small samples, this test should be used, since it produces fewer distortions than the chi-square test.

The sample was only gathered after the theme enlightening and authorization of the individuals. Anonymity as well as the confidentiality of the data obtained were guaranteed.

All subjects granted their informed consent for inclusion before participating in the study. The study was conducted in accordance with the Declaration of Helsinki, and the protocol was approved by the Ethics Committee of the Secretária Municipal de Saúde, Santo André (1.587.630).

## 3. Results

### 3.1. First Phase

Questionnaires were distributed with the purpose of establishing a social, demographic and biographic profile of the location. Table 1 shows the most significant variables of the community.

The several variables that were incorporated in the study and tabulated the appropriate statistical program mentioned above are the best ways of reflecting the social and demographic landscape of the target population. It is important to note that the variable “Sanitation” in Table 1 corresponds to the presence of the official public water, sewage and electricity networks.

As can be seen by observing Table 1, there is a predominance of females and the age groups from 19 to 30 and from 31 to 50 are the most prevalent in respondents. It was also verified that the income was below two Brazilian minimum wages (M.W.), which indicates that we face a special group in terms of economic capacity. It is also noted that there is a prevalence of masonry houses (63.1%) and houses with in-door plumbing/sanitation (64.3%).

### 3.2. Second Phase

In order to obtain plausible conclusions to the case study, it was necessary to compare the effects of the convenience sampling of another group of people that were not exposed to the several harmful environmental factors (Non-Exposed Group) to those who were exposed (Exposed Group).

Of the 84 individuals described in Table 1, 32 were randomly chosen and were designated as the “Exposed Group”. The Non-Exposed Group was interviewed at the same time as they did the blood sample. In total, 30 people were interviewed, which corresponds to the amount of blood samples.

As described earlier, in order for the importance of the two groups to share the same characteristics, it appears that the percentages are similar, where the female gender was the most prevalent, with 75% in the “Exposed Group” and 66.7% in “Non-Exposed Group”.

Regarding the age, the range between 31–50 years was the most commonly reported, with 11 people (36.7%) in the “Non-Exposed Group” and 19–30 in the “Exposed Group”, with 13 persons (40.6%).

It appears that the housing and financial realities are significantly different in each group, where 84.4% of the “Exposed Group” presents an income of less than two minimum wages, while 56.7% of the “Non-Exposed Group” have similar income.

Regarding the “Non-Exposed Group”, a greater purchasing power is observable, and, therefore, all of the respondents live in masonry houses and nearly all have sanitation. Table 2 describes the exposure type according to socio-biographic characteristics.

According to the analyzed variables, we observe that both groups have a significant association with the variables’ sewage (*p* = 0.004) house type (*p* < 0.0001) and income (*p* = 0.025) with the exception of the variables gender and age group (*p* > 0.05).

It is noted that of the totality of the individuals with sewage (82.3%), 94.4% of them live in the vicinity of the landfill.

According to house type, the totality of the non-exposed live in brick houses, and, from the total of individuals that live in this type (*n* = 50), the majority (62.5%) is exposed to the landfill.

The total of individuals that own an income lower than 2 M.W. is 44 (71%), as 27 of them (84.4%) are exposed to the landfill and (56.7%) correspond to the non-exposed.

In order to verify the association among hematologic changes towards socio-biodemographic characteristics, the next table was prepared (Table 3).

During the analysis of the hematologic changes, no significant association to any variables was observed.

Regarding the age group variable, among the age groups of 6–12 and 51–60, there is a great disparity among changes found. Overall, most (51.6%) do not show hematologic changes.

Regarding the sewage variable, there is a balance in terms of percentages, and we infer that (51.6%) do not have any hematologic changes. As regards the income variable, 44 individuals (71%) have an income lower than two minimum wages.

In order to investigate the existence of an association between exposure type and hematologic changes in individuals, a statistical analysis was carried out. The results of hemogram differences were summarized in Table 4.

In Table 4, we can observe an association among the variables of Exposure Type and Hematologic Change as being significant from a statistical perspective (*p* < 0.05). We verified a pattern of association between exposure and the presence/absence of hematologic changes (*p* = 0.022).

From the sum of people exposed to landfills (32 cases), the majority had hematological disorders (62.5%). It was found that, from 30 cases where the presence of hematological abnormalities was found, most are exposed to the landfill (66.7%).

As noted in Table 4, there was a 50% increase in changes in the screening performed in the “Exposed Group” compared to the “Non-Exposed Group”, which indicates that the population is not in its best health.

The chance of occurrence of hematological disorders is 3.33 times higher in subjects exposed to the landfill compared to those who presented hematological changes in the “Non-Exposed Group”. It is recognized, by the observation data, that 10 individuals from the “Non-Exposed Group” showed alterations in parameters, whereas the incidence in the “Exposed Group” was significantly higher, with 20 individuals undergoing changes.

Diseases such as leukocytosis, anemia, lymphocytosis and neutropenia were the major findings, and changes are evident in the “Exposed Group” compared to the “Non-Exposed Group”.

## 4. Discussion

Analyzing the changes in vital functions of living beings, it is possible to know the effects of exposure to pollutants before their occurrence and the existence of any other significant damages [18,23,24,25,26].

It is considered that proving the cause–effect connection with environmental exposures that may trigger chronic manifestations in humans requires specific studies that prove to be costly and time-consuming [27,28,29,30]. It is necessary for the data to be collected in the field so as to be compared with experimental observations, in order to demonstrate the process and how the interactions occur regarding study dynamics [16].

The effects of human exposure to environmental pollutants are manifested typically in the long term and are masked by other causes. Adding to this, the fact that the probability of harmful elements’ synergy and exponentiation of the risk is generally unknown, and it is thus extremely difficult to corroborate it with science based on laboratory tests, without incorporating other relevant factors, such as corporate interests, and industrial or professional regulators who hinder the analysis and detection of any effects on human health [20,24,26,30].

Given these considerations, as well as relying on the limitations of the health system itself in identifying peculiarities in the epidemiological profile of the population, a methodology was defined that aims to fully understand the exposure process. There are five parameters that are difficult to materialize including individual characteristics, duration of exposure, frequency of exposure, average time and “contact rate“, always bearing in mind that the characteristics of the individual vary by age, gender, occupation, and body weight [31].

The focus of the study is the exposure of individuals to organisms, as well as to social, economic and environmental processes in anticipation of models that historically focus on the monitoring and maintenance of health [21,22]. Several papers have been published that address the role that the landfill can play in people’s health [30,31,32]. There is no doubt that a landfill should be viewed from a holistic point of view, in that it must manage the direct and indirect effects that it may have [3,18,32].

Despite the existence of federal government programs for vulnerable populations, there do not seem to have been any improvements in the living conditions of this community [13,14]. The hemogram exam (counting blood cells—CBC) is used as a tool to promote the indicative tracking and respective improvements to minimize ongoing risks in analyses of a certain population. Given its great potential as a monitoring method, CBC integrates a set of parameters that describe the number and characteristics of some elements in the blood. The CBC consists of three basic features, namely erythrocyte evaluation (or red series), leukocytes evaluation (white or series), review of erythrocytes and platelets (platelet or series) [33].

Lymphocytosis is characterized by an increase in the number of lymphocytes (sub-group of white blood cells). The disease neutropenia indicates a low number of neutrophils that are a subset of leukocytes originating in the bone marrow, thus revealing an immune susceptibility. The increase of leukocytes is a sign of a viral infection, i.e., people near the landfill tend not to have viral and bacterial infections [33]. The disease leukocytosis reveals an increased number of leukocytes that reveals the existence of an infection, since they are the elements that are linked to immune system defenses against foreign bodies. The greater the leukocytosis is, the more contaminated the landfill will probably be, which can be interpreted as a parameter that reveals and establishes causal links. The disease of anemia is more complex, so the causes are more comprehensive and diversified. This immediately implies a more detailed investigation into these cases [33,34,35].

The lymphocytosis, neutropenia and leukocytosis diseases can be understood as correlated, although they have opposite directions of growth, as previously conveyed [35].

The results express the presence of discrepancies in the health of the São Jorge population, compared with the other subjects in the study, which requires the completion of further studies to detect the most common causes and the consequences for the residents in order to enable the development of a continuous and systematic supervision program for the residents. The suggestion is to carry out a more in-depth study in order to make improvements in this type of group exposed to potential health hazards, such as those identified in this study, in comparison with the Non-Exposed Group.

The problems derived from municipal solid waste are still present and without a proper solution [36]. There is no alternative but a behavioral change in relation to waste, given the reduction in its production, and in order to gradually implement technologies that are within our technical capabilities and to leverage resources to gradually develop a greater level of control over the environmental and health effects caused by waste [37].

The prevention measures and control of public health consequences of urban solid waste lack information and epidemiological data in which causal relationships can be established. There is a colossal deficiency in studies on the recovery of areas degraded by disposal of urban solid waste. In this context, this article seeks to contribute to a consolidation of the state-of-the-art related to the theme, in order to raise awareness of the elements that improve the quality of urban areas and hence the quality of life of their citizens [31,36].

Supporting research of this kind is a priority. The development of greater technical training, in view of the environmental and health issues, as well as the involvement of professionals in integrated waste management systems, in the medium and long term, may introduce these variables in projects and plans [15,37,38].

Data from this study should be compared to other data, which may involve a characterization of the epigenetic profile of the population [39] and even a characterization of the study areas for the presence of particulate material [40]. The challenge is to continue collecting more valid and reliable data in order to achieve an extrapolation between environmental and respective consequences for the health of population’s risk factors to make a comparison on a national and global scale [18]. The study described is the first step in a study involving the whole *Espírito Santo* community.

The data obtained in this tracking study indicate that strategies for prevention and health promotion should include joint actions established between citizens and the management of services and should always be aimed at improving living conditions, particularly urban planning, implementation educational programs, as well as raising awareness about behavioral changes, once isolated actions are considered ineffective in reducing any problems [38,41].

It can be inferred that this study has a purpose of consolidating knowledge, which contributes to the prevention and detection of either known or unknown adverse health effects [40,41] to the public that are related to environmental exposure. The study should be understood as a primary screening indicating some pathologies, which, in addition to enabling us to assess the state of health of the inhabitant, may be indicative of the level of pollution of landfill, insofar as the conditions are reflected on the inhabitants of the landfill.

We must identify priorities at the community level, which is why the understanding of environmental health problems is essential. Sustainable planning may be a possible tool to implement a research-oriented future intervention, and thus to address the issues of greatest importance. However, this process is not simple in communities featuring persistent health disparities and a historical lack of confidence in health professionals [40,41].

The main alterations found in the completed study were anemia, leukocytosis, lymphocytosis and neutropenia. These data show that this is a group with greater immune susceptibility, adding that some elements of the exposed group already have infections, which are consistent with exposure to contaminants, supporting the existence of a pattern. Aiming at the promotion of health, it is suggested that the project should continue to be monitored in order to gather a greater quantity of evidence in support of the result of the tracking work carried out and described [42,43].

Based on the study, the exposed group is 3.33 times more likely to develop hematological abnormalities, taking as reference its exposure to the landfill, as opposed to the group that was not exposed. There is much [8,44] research on urban landfills, but none involving the social and hematological profile have been found in the literature.

Although the results only show the events occurred at the São Jorge landfill, in the Greater ABC region. They also provide important information on the expansion of knowledge concerning the assessment of this risk in residential areas around the planet. The demand for a thorough investigation is urgent in order to spread information related to the potential effects of human exposure to contaminants from multiple sources that affect public health.

A recent bibliographical survey study about the difficulty of assessing the impacts on the health of populations exposed to waste [45] shows that the biggest difficulty is the access to immediate and recent results of studies about biomonitoring of the effects of waste exposure on human health [45]. It is noted that this occurs due to several factors: identifying the profile of the exposed population, characterization and diversity of the analyzed area, socio-economic factors (education, unemployment, home ownership, family structure and access to health services), as well as government interests.

The analysis provides evaluation of quantitative data on the environment. However, the complexity and specificity related to historical and cultural features of this population caused some limitations in this study, such as: it was a convenience sampling, the way people moved to the landfill area, and, mainly, whether these blood changes in people living near the waste were due to their displacement alone. Besides those, there are limited potential confounders, such as population diet, family history and other risk factors that were not controlled in the analyses.

Future investigations may include concepts like nanoparticles and epigenetics, in order to promote a holistic view of the causes, mechanisms and consequences caused by the exposure of humans to a multitude of organisms, thereby complementing the present study and scientific knowledge, eliminating existing cross-limitations.

## 5. Conclusions

The full blood element count analysis was performed and it found that the blood counts of residents living near the landfill had positive results for hematological changes, and diseases such as leukopenia, anemia, neutropenia and lymphocytosis were the most frequently encountered changes. However, proving the cause-effect relation with environmental exposure factors that may trigger chronic manifestations in humans requires specific studies, which often are very costly and time-consuming.

## Figures and Tables

**Table 1:** Socio-biodemographic characteristics of the inhabitants.

Variables	Community Sample	
	*n*	%
Gender		
Female	55	65.5
Male	29	34.5
Age group		
(6–12)	7	8.3
(13–18)	6	7.1
(19–30)	30	35.7
(31–50)	31	36.9
(51–60)	8	9.5
(+60)	2	2.4
House type		
Bricks	53	63.1
Wood	20	23.8
Mix	5	6.0
Another	6	7.1
Income *		
Less than 2	67	79.8
Between 3–5	16	19.0
Between 5–10	1	1.2
Plus 10	0	0.0
Sanitation		
Yes	54	64.3
No	30	35.7
Total	84	100

* Minimum Wage (M.W.) = 724 Reais.

**Table 2:** Association of the socio-biodemographic characteristics with the exposition type.

Socio-Biographic Characteristics		Exposition Type						*p*-Value
		Non-Exposed		Exposed		Total		
		*n*	%	*n*	%	*n*	%	
Gender	Female	20	66.7	24	75	44	71	0.471
	Male	10	33.3	8	25	18	29	
	Total	30	100	32	100	62	100	
Age Group	(6–12)	2	6.7	6	18.8	8	12.9	0.309
	(13–18)	1	3.3	3	9.4	4	6.5	
	(19–30)	9	30	13	40.6	22	35.5	
	(31–50)	11	36.7	6	18.8	17	27.4	
	(51–60)	5	16.7	3	9.4	8	12.9	
	≥60	2	6.7	1	3.1	3	4.8	
	Total	30	100	32	100	62	100	
Sanitation	Yes	29	96.7	22	84.4	51	82.3	0.004 *
	No	1	3.3	10	15.6	11	17.7	
	Total	30	100	32	100	62	100	
Type of House	Brick	30	100	20	62.5	50	80.6	<0.001 *
	Wood	0	0	12	37.5	12	19.4	
	Total	30	100	32	100	62	100	
Income	Less 2 M.W.	17	56.7	27	84.4	44	71	0.025 *
	Among 3–5 M.W.	9	30	5	15.6	14	22.6	
	Among 5–10 M.W.	4	13.3	0	0	4	6.5	
	Total	30	100	32	100	62	100	

* Statistical significance (chi-square test; exact Fisher).

**Table 3:** Hematologic changes discovered in individuals’ hemograms.

Variable	Description	Hematologic Changes						*p*-Value
		Yes		No		Total		
		*n*	%	*n*	%	*N*	%	
Gender	Female	20	45.5	24	54.5	44	29.0	0.470
	Male	10	55.6	8	44.4	18	71.0	
	Total	30	45.5	32	54.5	62	100	
Age Group	(6–12)	5	62.5	3	37.5	8	12.9	no assumptions
	(13–18)	2	50.0	2	50.0	4	6.5	
	(19–30)	11	50.0	11	50.0	22	35.5	
	(31–50)	9	52.9	8	47.1	17	27.4	
	(51–60)	2	25.0	6	75.0	8	12.9	
	≥60	1	33.3	2	66.7	3	4.8	
	Total	30	48.4	32	51.6	62	100	
Sanitation	Yes	25	49.0	26	51.0	51	82.3	no assumptions
	No	5	45.5	6	54.5	11	17.7	
	Total	30	48.4	32	51.6	62	100	
House Type	Masonry	25	50.0	25	50.0	50	80.6	0.604
	Timber	5	41.7	7	58.3	12	19.4	
	Total	30	48.4	32	51.6	62	100	
Income	Less 2 M.W.	17	56.7	27	84.4	44	71	no assumptions
	Between 3–5 M.W.	9	30	5	15.6	14	22.6	
	Between 5–10 M.W.	4	13.3	0	0	4	6.5	
	Total	30	100	32	100	62	100	

**Table 4:** Association among exposure type with hematologic change.

Groups			Hematologic Change		
			Change	No Change	Total
Exposure Type	Non-Exposed	*n*	10	20	30
		% Line	33.3%	66.7%	100.0%
		% Column	33.3%	62.5%	48.4%
		% Total	16.1%	32.3%	48.4%
	Exposed	Count	20	12	32
		% Line	62.5%	37.5%	100.0%
		% Column	66.7%	37.5%	51.6%
		% of Total	32.3%	19.4%	51.6%

*p* = 0.022.
